# Molecular flexibility of hyaluronic acid has a profound effect on invasion of cancer cells

**DOI:** 10.1098/rsos.251036

**Published:** 2025-08-27

**Authors:** Uliana Bashtanova, Agne Kuraite, Rakesh Rajan, Melinda Duer

**Affiliations:** ^1^ Yusuf Hamied Department of Chemistry, University of Cambridge, Cambridge, UK

**Keywords:** hyaluronic acid, extracellular matrix, nuclear magnetic resonance spectroscopy, molecular dynamics, glioblastoma

## Abstract

Extracellular hyaluronic acid (HA) has been shown to be important in cancer; low-molecular-weight HA typically correlates with cancer progression, high-molecular-weight HA with homeostasis. Here we show that even high-molecular-weight HA can induce cancer cell migration when it is highly diluted. HA-induced cell signalling is primarily through HA binding to the cell surface receptor, CD44. We show by NMR spectroscopy that at high dilution, high-molecular-weight HA molecules access the conformations needed for strong binding to CD44 on the tens of nanosecond time scale, the relevant time scale for induction of CD44 signalling. We further show that, by contrast, at higher concentrations, high-molecular-weight HA molecules have insufficient flexibility for strong CD44 binding. The high dilution HA condition correlates with profound changes in brain cancer cell morphology and proteome which supports cancer cell invasion. We hypothesize that the flexibility of HA molecules is central to HA-mediated cell signalling and that this concept can explain previous observations that the outcome of HA-mediated signalling depends on the HA molecular weight. HA dilution leading to stronger HA signalling may be important in understanding the role that oedema plays in cancer recurrence after primary surgery.

## 1. Introduction

Glioblastoma (GBM) is the most common brain cancer and has very poor prognosis, with median patient survival time of less than 15 months. The main reason for such poor survival times is the diffuse infiltration by GBM tumours of the brain parenchyma, and invasion of cancer cells through perivascular and perineuronal spaces. Throughout these processes, the tumour cells encounter one of the most abundant components of the brain extracellular matrix: hyaluronic acid (HA), also known as hyaluronan and hyaluronate. In gliomas, HA concentration is even higher than in normal brain tissue [1] and so is a major component of the tumour microenvironment (TME). HA degradation is an additional consistent feature of GBM [2]. The HA-degradation enzyme, hyaluronidase 2 (HYAL2) is over-expressed in GBM and HYAL2 expression negatively correlates with patient survival time [3]. Collectively, these studies suggest that lower-molecular-weight HA facilitates disease progression in GBM.

Studies in other cancers have also collectively led to the view that low-molecular-weight HA (LMW-HA; molecular mass 10–250 kDa) plays significant roles in cancer progression [4–8]. By contrast, high-molecular-eight HA (HMW-HA; molecular mass >500 kDa) has more commonly been found to support normal tissue homeostasis and inhibit tumour formation. However, there are numerous exceptions to these trends [5,6,8], and considerable conflict remains about the mechanisms behind apparent differences in cancer cell behaviour in the presence of HA of different molecular weights. Given the prevalence of HA in the TME [3] in GBM, it is important to understand the mechanism behind any GBM progression-dependence on HA molecular weight.

**Figure 1.**
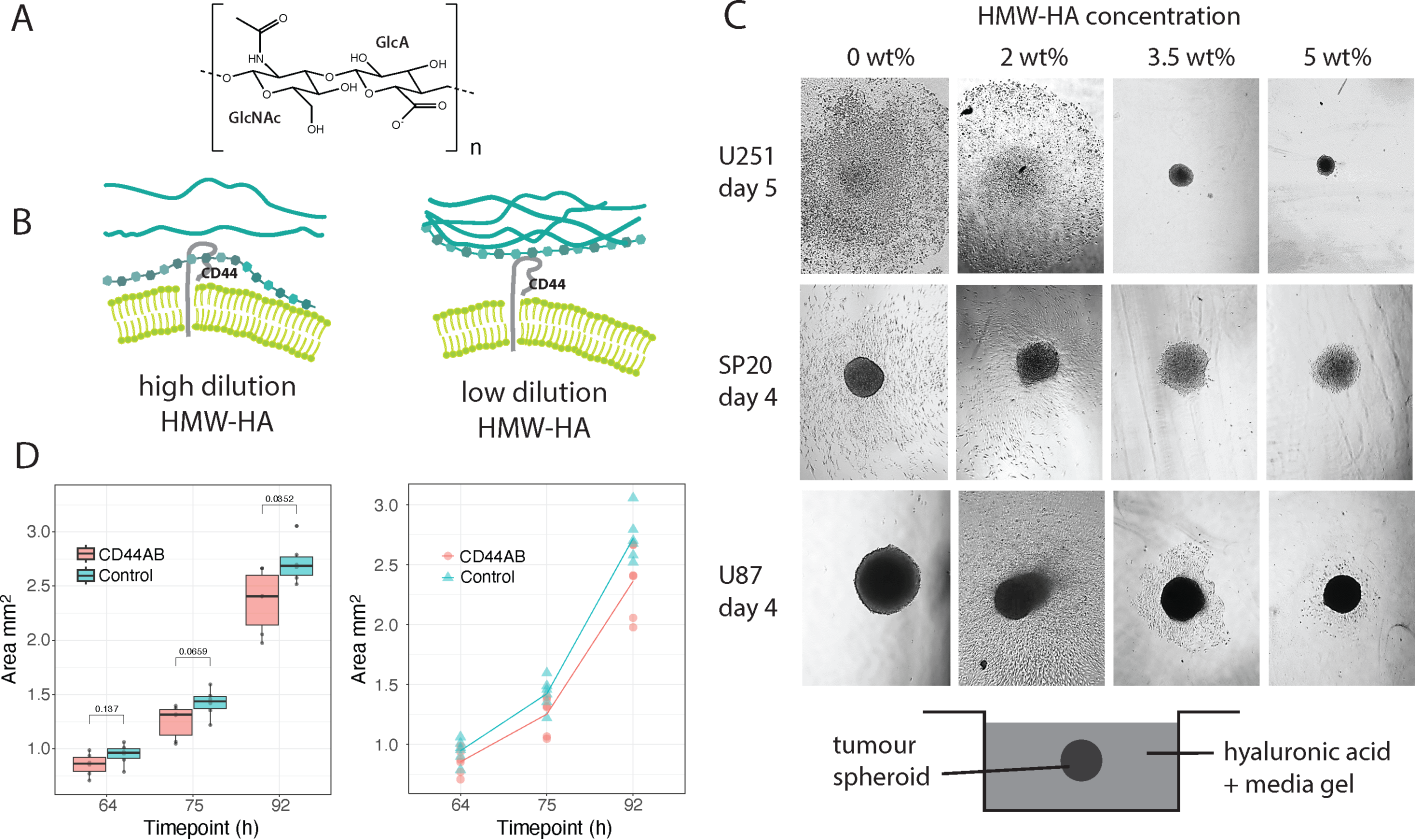
Cancer spheroid behaviour in different HMW-HA concentrations. (A) Chemical structure of HA. HA is a polymer of variable length, *n*. (B) Schematic illustrating that lower concentrations of HMW-HA have lower density of molecular entanglements and intermolecular electrostatic interactions, and thus greater molecular flexibility (greater motional degrees of freedom). (C) Spheroids were grown in media for a period of 7 days, after which multiple spheroids were embedded in each concentration of HMW-HA gel. Representative microscope images of cell migration from the spheroids after 4 or 5 days of culture in different HA concentrations illustrate typical spheroid behaviour in each specific HMW-HA concentration. Field view 1900 µm x 2500 µm. (D) Area of cell migration from U87 spheroids in 2 wt% HMW-HA as a function of time for spheroids pre-incubated with polyclonal antibody to CD44 (CD44AB) compared with controls with no antibody.

HA is a polymer with a simple, repeating disaccharide structure (figure 1A), so both long (HMW-HA) and short (LMW-HA) molecules have the same chemical composition. Thus it is intriguing that HA being a long or short polymer can apparently have such contrasting effects on cancer progression. We reasoned that the primary difference between long and short HA molecules is their respective molecular flexibility. The degree of molecular flexibility can be expected to affect on-off binding rates of HA with its cell surface receptors, and so impact cell signalling and hence tumour progression. This would have particular relevance in GBM, given the high concentration of HA in the TME and thus the potential for activation of HA signalling pathways in cancer cells [9–13].

HA flexibility is determined in part by the extent of molecular entanglement with other surrounding polymeric molecules (figure 1B). Shorter HA molecules have lower entanglement than longer HA molecules and so have greater flexibility for the same HA monomer concentration. The other factor affecting HA molecular flexibility are the intermolecular interactions with surrounding molecules (figure 1B). For a highly hydrophilic polymer like HA, intermolecular HA–HA interactions are mediated via the intervening water network. The larger the water network, the weaker the effective binding between HA molecules. Thus, the effect of both intermolecular interactions and polymer entanglement is to increase molecular flexibility with decreasing HA concentration. We reasoned that if HA molecular flexibility is the important parameter in determining cancer cell behaviour, then even HMW-HA could promote cancer progression if it is sufficiently diluted with water that it attains the necessary degree of molecular flexibility to initiate cell signalling.

## 2. Results

### 2.1. Low concentration HMW-HA promotes cancer cell migration as effectively as LMW-HA

To test the hypothesis that HMW-HA can promote cancer cell migration if sufficiently dilute, we generated 3D spheroids from two cancer cell lines of neuroepithelial origin (U251 and U87) and from patient-derived SP20 tumour-initiating cells isolated from glioblastoma tumour mass after surgical resection [14], and we placed these spheroids in a range of HMW-HA concentrations [15]. We found that for all cell types, invasion into 2 wt% HMW-HA was very advanced by day 4 (figure 1B), but only a few cells were dispersed into 5 wt% HMW-HA (figure 1B) and their quantity did not change for periods of up to 6 weeks. In all cases, the spheroids remained suspended in the gel^1^ (electronic supplementary material, figure S3A) and cell migration was through the HA gel, not along the bottom of the well plate, except for spheroids in the media-only controls. One might argue that the greater cell migration in relatively low concentrations of HMW-HA could be an effect of lower viscosity HA gels presenting lower resistance to cell migration. However, in control liquid media, in which viscosity was comparable with viscosity of water, so three orders of magnitude smaller than the HMW-HA gels, cancer spheroids demonstrated variable behaviour: from a full dispersion of U251 cells, intermediate for SP20 and almost none for U87 cells (figure 1C). We performed the same experiments for the U87 cell line in LMW-HA (electronic supplementary material, figure S4). Considerable cell migration was observed by day 2 in both 2 and 5 wt% LMW-HA concentrations (electronic supplementary material, figure S4), similar in extent to that for spheroids in 2 wt% HMW-HA.

Thus, for the U87 cell line (but not the U251 or SP20 cell lines), the observed invasion was clearly not due to the viscosity of the environment. This is evident from the absence of invasion at 0 wt% HMW-HA, strong invasion at 2 wt% HMW-HA, and inhibition of invasion at 5 wt% HMW-HA. Additionally, invasion was not driven by the molecular mass of the HA polymer, as LMW-HA supported invasion at both tested concentrations, similar to 2 wt% HMW-HA, while 5 wt% HMW-HA inhibited invasion.

These findings prompted us to explore three key questions about U87 cell invasion in HA-containing gels, independent of viscosity and polymer length. First, is invasion in 2 wt% HMW-HA specifically mediated by interactions between HA and its cellular receptors? Second, could the molecular flexibility of HA be a critical factor, permitting invasion at 2 wt% HMW-HA but hindering it at 5 wt%? Third, might differences in the expression of HA receptors or hyaluronidases account for the contrasting invasion behaviour between 2 wt% and 5 wt% HMW-HA?

### 2.2. HA-CD44 binding promotes cell migration

We first investigated whether the invasion in 2% HMW-HA was due to HA interaction with its cellular receptor. The primary cell surface receptor for HA is CD44 [10,16], and CD44-HA interactions have been shown to be important in determining cancer cell behaviour *in vitro* [17–19]. We hypothesized that the flexible HMW-HA molecules in 2 wt% gels could promote HA binding to CD44 compared with the more restricted molecules in 5 wt% gel (figure 1C) and that this binding would promote cell migration.

We first tested whether CD44 was the specific receptor of HMW-HA responsible for promotion of U87 cell migration. Search for HA receptors in our proteomic studies (see below) yielded only one receptor—CD44, and as expected for a chemoreceptor, CD44 was upregulated with increasing HMW-HA concentration (see §2.4). We then blocked CD44 on the surface of U87 spheroids with a polyclonal CD44 antibody, and placed the spheroids in 2 wt% HMW-HA gel (figure 1A,D). Although there are many cell surface receptors involved in cell migration, we expected that blocking CD44 sites would nevertheless retard cell dispersion from spheroid, if HMW-HA binding to CD44 is relevant to U87 cell migration.

We observed a steady trend towards smaller migration areas in spheroids with blocked CD44 compared with control, resulting in statistically smaller migration areas by 92 h (figure 1D) confirming that CD44 binding to HMW-HA was an important component of U87 cancer cell migration behaviour.

### 2.3. HMW-HA molecular flexibility in 2 wt%, but not 5 wt% gels is sufficient for strong CD44 binding

**Figure 2.**
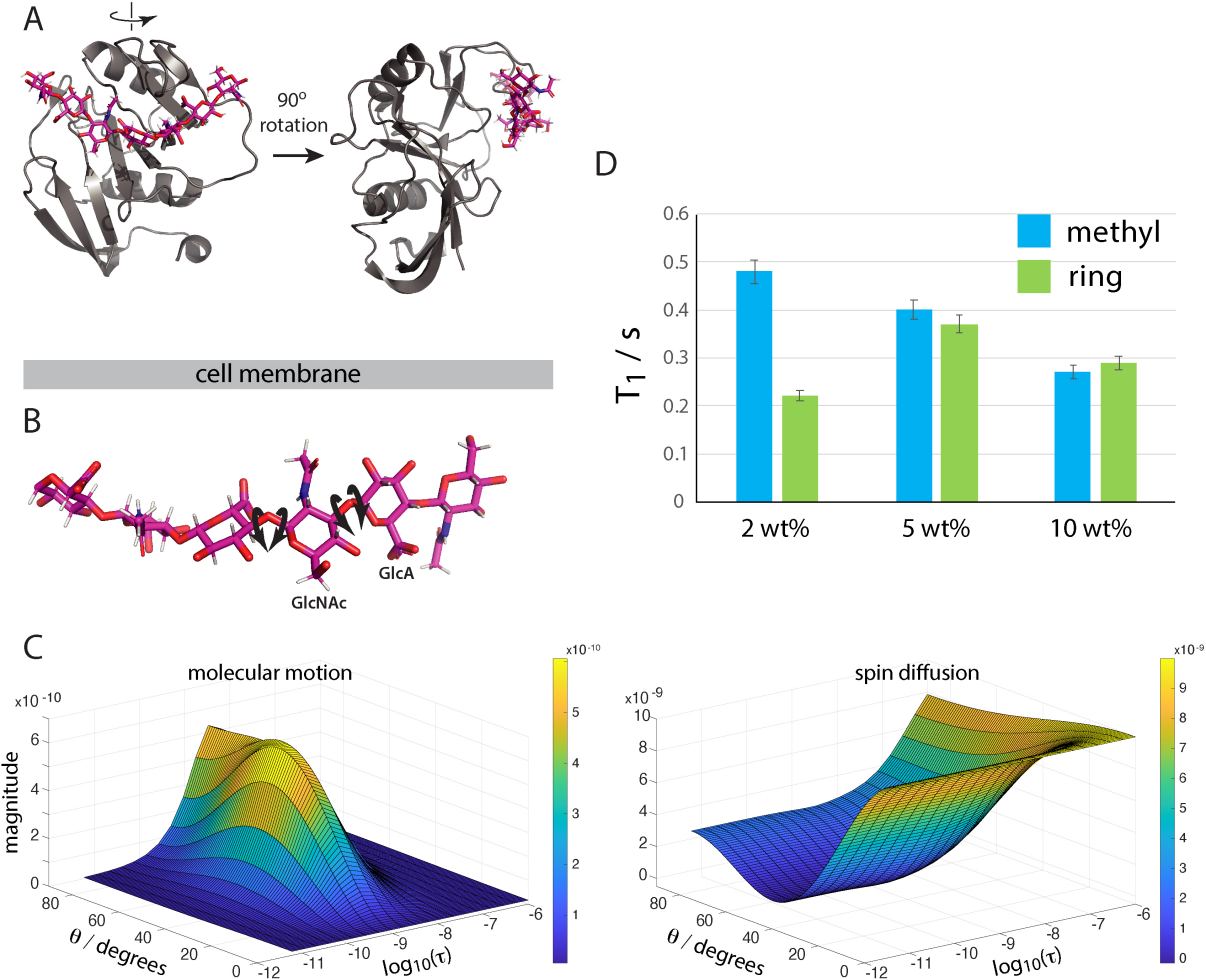
(A) Type A binding of HA 8-mers on the CD44 hyaluronic acid binding domain (HABD) (from PDB 2JCQ [20]). The HA molecule must contort its conformation in order to access the HABD binding site in this ligand binding mode, and similarly in type B ligand binding (not shown, PDB 2JCR [20]). ‘Cell membrane’ indicates the approximate orientation of the HABD relative to the cell plasma membrane. (B) Molecular structure of HA in its extended state (3HYA), illustrating the HA internal molecular motions around the glycosidic bonds that contribute to ^1^H spin-lattice relaxation. Motions that change the orientation of ^1^H^–1^H vectors can potentially contribute to ^1^H T_1_ for HA. Carbon atoms: pink; oxygen: red; nitrogen: blue; hydrogen: white. (C) The magnitudes of the contributions of internal HA molecular motions with correlation time *τ* and spin diffusion in the presence of isotropic molecular tumbling (with correlation time *τ*_macro_ = 10^−7^s here; see electronic supplementary material, figure S4 for simulations with other *τ*_macro_) to ^1^H T_1_ See electronic supplementary material, text S1 for simulation details. The internal motion is random ^1^H–^1^H vector motion on a cone of cone angle *θ*. The simulation shows that the molecular motion term dominates over the spin diffusion term only for *t* between approximately 10^–8^ s and 10^−10^ s and angular amplitudes of motion above approximately 30^o^. (D) Experimental ^1^H T_1_ (see electronic supplementary material, text S2 for definition) for HMW-HA ring (non-OH) and GlcNAc methyl ^1^H as a function of HA concentration. See electronic supplementary materials, text S3 for details of experimental T_1_ data analysis, table S2 and figure S7 for fitted data.

We asked, can the increased flexibility of HMW-HA in 2 wt% gel promote CD44-HA binding compared with 5 wt%? Structural studies have shown that HA-CD44 binding requires both HA and the CD44 HA binding domain (HABD) to adopt specific conformations [20–22] (figure 2A). Previous analysis of crystal structures for free and CD44 HABD-bound HA showed that the conformation of the portion of HA in the postulated strong-binding mode [20] (figure 2A) was significantly distorted from the ground-state structures predicted to exist in bulk HA (figure 2B) [23–27], with a kink of around 22° in the HA molecular long axis in the HABD (see electronic supplementary material, figure S5) [20]. Molecular dynamics studies suggested that the process of adapting the combined molecular conformations for strong binding was of order of tens to hundreds of nanoseconds [28–30].

Based on these studies, we hypothesized that the molecular flexibility of HMW-HA in 2 wt% gel allowed the HA molecule to access conformations that fit the HABD for strong binding on the tens–hundreds of nanoseconds time scale, triggering signalling that promotes cell migration. We also hypothesized that such conformational distortions were exceptionally rare for restricted HMW-HA molecules in 5 wt% gel, and hence signalling events that promote cell migration were also exceptionally rare.

To test these hypotheses, we next used nuclear magnetic resonance (NMR) ^1^H spin-lattice relaxation time measurements to test whether HMW-HA molecules in 2 wt% gel meet the criterion for strong HABD binding of more than 22° amplitude backbone fluctuations with nanosecond correlation times, and conversely to check that in 5 wt% gel, HMW-HA molecules do not meet this criterion. NMR ^1^H spin-lattice relaxation rates for water-binding polymers like HA depend on three terms: (i) molecular conformation changes or rotations on the nanosecond time scale; (ii) cross-relaxation between the dipole-coupled ^1^H in the polymer through spin diffusion; and (iii) ^1^H exchange between exchangeable ^1^H in the polymer and surrounding water molecules [31–34] (see electronic supplementary material, text S1 for equations and text S2 for details of effect of term (iii)).

In high concentration, polymer networks where motion of individual molecules with nanosecond correlation times is strongly restricted, term (i) is small, and the cross-relaxation of term (ii) is expected to dominate (see electronic supplementary material, text S1, equation (1)). This scenario is identified by all the observed polymer ^1^H signals having the same relaxation times. By contrast, where individual polymer molecules have molecular conformational flexibility with nanosecond correlation times, term (i) makes a significant contribution and term (ii) is quenched (because the ^1^H–^1^H dipolar coupling that drives this term is partially averaged by the molecular motion). In this scenario, the ^1^H relaxation times depend on the amplitudes of nanosecond time scale motions of the different polymer functional groups containing the ^1^H.

To quantify the motion correlation times and angular amplitudes that result in the ^1^H spin-lattice relaxation rate molecular motion term (i) dominating over the spin diffusion term (ii), we modelled how the size of these two terms vary with motional correlation time and amplitude. The dominant interaction leading to ^1^H relaxation in the HA system is ^1^H–^1^H dipolar coupling. We modelled the case for random motion of a ^1^H–^1^H dipolar coupling vector on a cone of cone angle *θ* (amplitude of the molecular motion). The molecular motion of the HA rings (figure 2B) and side groups in an aqueous gel is clearly highly complex. There will be a spectrum of motional modes about different bonds and molecular axes, each of which can have different amplitudes and correlation times. Our aim is to understand the magnitude of the HA backbone angular amplitudes of motion and correlation times that would lead to the molecular motion term governing HA ^1^H relaxation to dominate over the spin diffusion term. Hence, we chose this simple model of molecular motion, knowing that the true molecular motions can be considered as made up of contributions from similar simple motions about (many) individual bonds and molecular axes that result in the same overall conformational fluctuations. The results from our model in figure 2C show that the spin diffusion term has a maximum value that is roughly an order of magnitude larger than the molecular motion term and thus will always dominate unless the molecular motion meets the conditions close to maximizing the molecular motion term; that is (from figure 2C) correlation times between 10^−8^ s and 10^−10^ s and angular amplitudes of motion above approximately 30° (i.e. for amplitudes of motion consistent with the HA backbone accessing molecular conformations able to fit the CD44 HABD).

For our HA gels, we experimentally measured ^1^H spin-lattice relaxation times for the sugar ring ^1^H and acetyl methyl group ^1^H (see electronic supplementary material, text S3). From the above discussion, HA having sufficient amplitude of backbone molecular motion with nanosecond correlation times for strong binding to the CD44 HABD would be indicated by methyl and sugar ring ^1^H having different spin-lattice relaxation time constants, T_1_ (see electronic supplementary material, text S2).

Figure 2D shows the ^1^H T_1_ determined at 37°C for HMW-HA methyl groups in the N-acetyl-D-glucosamine rings and the non-exchangeable sugar ring ^1^H collectively for HA dilutions of 10, 5 and 2 wt% in cell culture medium (see electronic supplementary materials, text S3, figure S6 and table S2 for analysis of experimental T_1_ data). The ^1^H T_1_ were the same within error for methyl and ring ^1^H at the higher HMW-HA concentrations (5, 10 wt%), indicating that ^1^H cross relaxation, spin diffusion, is the dominant term governing HA ^1^H spin-lattice relaxation at these higher concentrations, and thus that only low amplitude HA backbone fluctuations occurred on the nanosecond time scale. By contrast, the methyl and ring ^1^H T_1_ were significantly different to each other in 2 wt% HMW-HA, showing that HA molecular motions on the nanosecond time scale now governed the ^1^H T_1_, and thus that the HA backbone had motions that included significant fluctuations with amplitudes greater than approximately 30°. These data were consistent with rheology measurements on the HMW-HA gels (electronic supplementary material, figure S8) which showed that the crossover oscillation frequency for the storage and loss moduli which represented the intrinsic molecular disentanglement rate was an order of magnitude higher for the 2 wt% gel compared with 5 wt%. For comparison, we also measured ^1^H T_1_ values for LMW-HA at the same 2 and 5 wt% concentrations (electronic supplementary material, table S2). The HA ring and methyl ^1^H T_1_ time constants were significantly different to each other for both LMW-HA concentrations, again consistent with rapid molecular motions on the nanosecond time scale (see electronic supplementary material, table S2 for further details).

Thus, we concluded that HMW-HA flexibility on the nanosecond time scale was a controlling factor for CD44 binding, thus mediating cell signalling, and influencing cell migration or its inhibition.

### 2.4. Expression of structural and signalling proteins in U87 cancer cells underpins their behaviour in 2 versus 5 wt% HMW-HA

**Figure 3.**
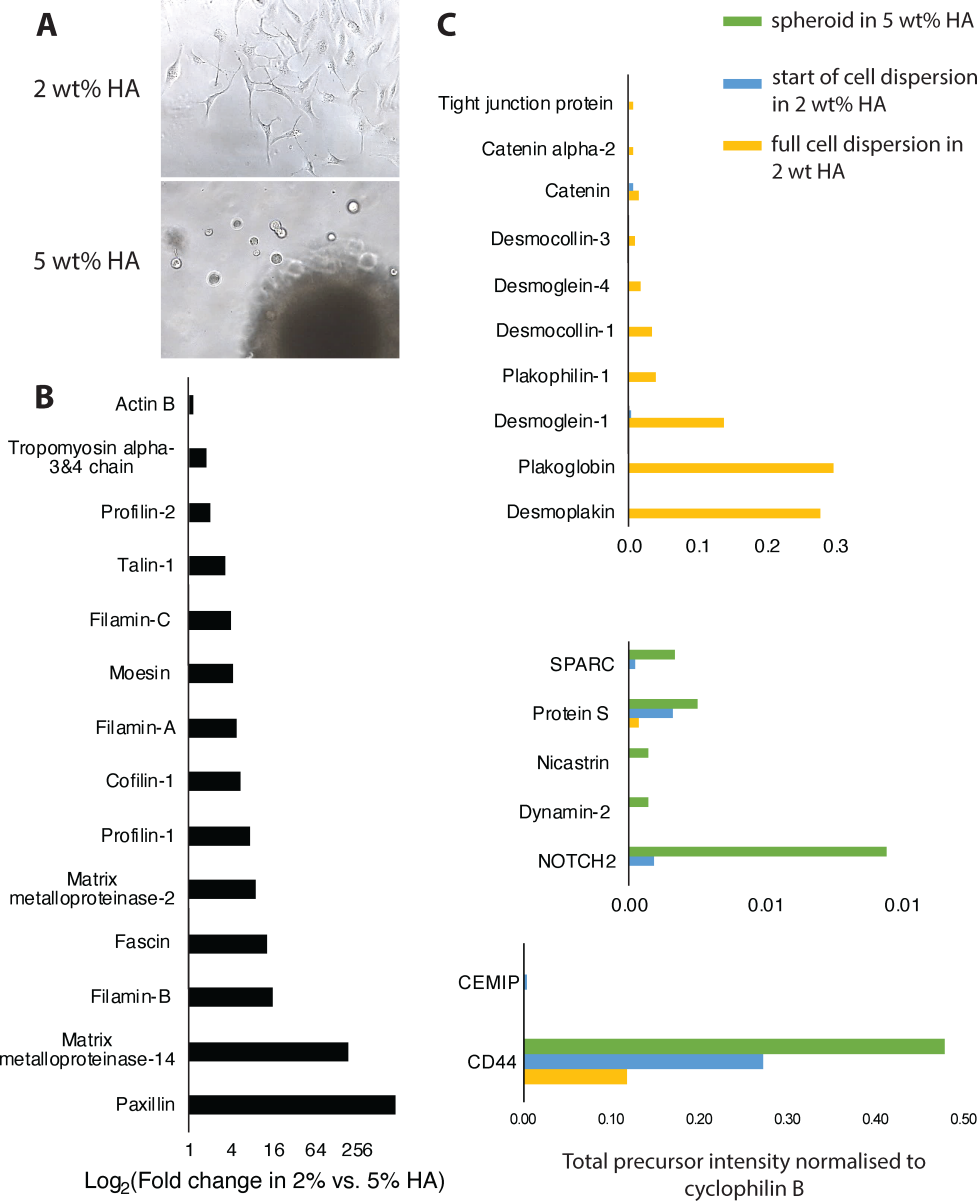
Changes in U87 proteome relevant to changes in cell morphology and behaviour in HMW-HA. (A) Morphology of U87 cells dispersed from spheroid in HMW-HA. Field view 1900 µm × 2500 µm. (B) Upregulation of invadopodium proteome in 2 wt% HMW-HA after full cell dispersion from spheroid compared with spheroid in 5 wt%. (C) Changes in cell–cell junction proteome and CD44/Notch axis in different HMW-HA concentrations and at two different stages of cell dispersion in 2 wt% HMW-HA.

To gain some understanding of how HMW-HA flexibility controls U87 cell behaviour and probe the question: might differences in the expression of HA receptors or hyaluronidases account for the contrasting invasion behaviour between 2 wt% and 5 wt% HMW-HA, we utilized a proteomic approach. We had observed that not only was cell migration drastically different between 2 wt% and 5 wt% gels (figure 1C), but cell morphology was also different: in 5 wt% HMW-HA cells were typically rounded and lacked invadopodia, but in 2 wt% HMW-HA cells adopted star-shape flattened morphologies with very long and branching invadopodia (figure 3A; electronic supplementary material, S3B). We reasoned that the radical difference in cell morphology as well as migration behaviour in 2 versus 5 wt% HMW-HA had to be underpinned by widespread changes in protein biosynthesis.

Shotgun label-free proteomics is widely used in discovery studies. For our pilot study, we used it in a semi-quantitative mode (i.e. without extensive replicas), so it was important to minimize any contributions to variability unrelated to change in HMW-HA concentration. The contribution to variability in human brain samples was previously assessed by Piehowsky *et al.* [35] and rated as follows: sampling of biological tissue (72%) was much greater than instrumental variance (i.e. short-term run-to-run instrumental response fluctuation) (16%), which in turn was greater than instrumental stability (i.e. long-term drift of the quantitative response of the LC–MS/MS platform over a 2 week period) (8.4%), which was greater than digestion (3.1%) [35]. However, for *in vitro* cell cultures it was shown that total coefficient of variation (CV) was much lower compared with tissues: CV for bacterial culture was 11% [36] compared with CV = 34% for *ex vivo* human brain samples [35]. Thus, using *in vitro* grown U87 cancer cells permitted implementation of semi-quantitative approach to compare protein expression in samples grown at different HA concentrations with the caveat that all precautions were taken to minimize variability arising from instrumentation, sampling and protein extraction and digestion (see Experimental Methods). Furthermore, in our analysis we decided to rely on directional changes in groups of structurally co-operating proteins, for example, in the invadopodium scaffold proteome and cell–cell junction proteome.

To corroborate this approach, we first analysed the group of invadopodium scaffolding proteins, because this cellular structure was clearly present in 2 wt% HMW-HA but absent in 5 wt% HMW-HA. We found that key invadopodium scaffold proteins were all upregulated by 2 to 200-fold in 2 wt% compared with 5 wt% HMA-HA (figure 3B). Foremost upregulated were proteins that structurally reorganize actin into stable filaments and bundles (figure 3B) such as fascin-1, which ensures actin bundle stability by binding to F-actin at regular intervals [37], filamins A and C, known to be involved in non-covalent cross-linking of F-actin [38], and tropomyosin 3 and 4, as a master regulator of individual F-actin filament function, with isoforms 3 and 4 most characteristic for invasive cancers [39]. Proteins implicated in early stages of invadopodium formation were also upregulated (figure 3B), including profilins which promote actin assembly when barbed ends are free [40] and cofilin-1 which drives membrane protrusion by severing actin filaments and their directional extension by treadmilling [41]. Also upregulated were three key proteins, which connect the internal actin cytoskeleton to adhesion receptors on plasma membrane (figure 3B), in particular moesin which links actin filaments to CD44 [42], and talin and paxillin which are essential for activation of integrins [43], recruitment of phosphatases and kinases and their interconnection with actin bundles [44]. The invadopodium is considered mature when it recruits matrix metalloproteases (MMPs) [45], and in 2 wt% HMW-HA, MMP2 and MMP14 were highly upregulated compared with 5 wt% HMW-HA (figure 3B). Expression of β-actin did not change in different HMW-HA concentrations, as expected (figure 3B), because β-actin is normally reorganized in different environments rather than regulated by biosynthesis [46] and hence widely used as an internal standard and ‘housekeeping control’ in protein studies.

**Figure 4.**
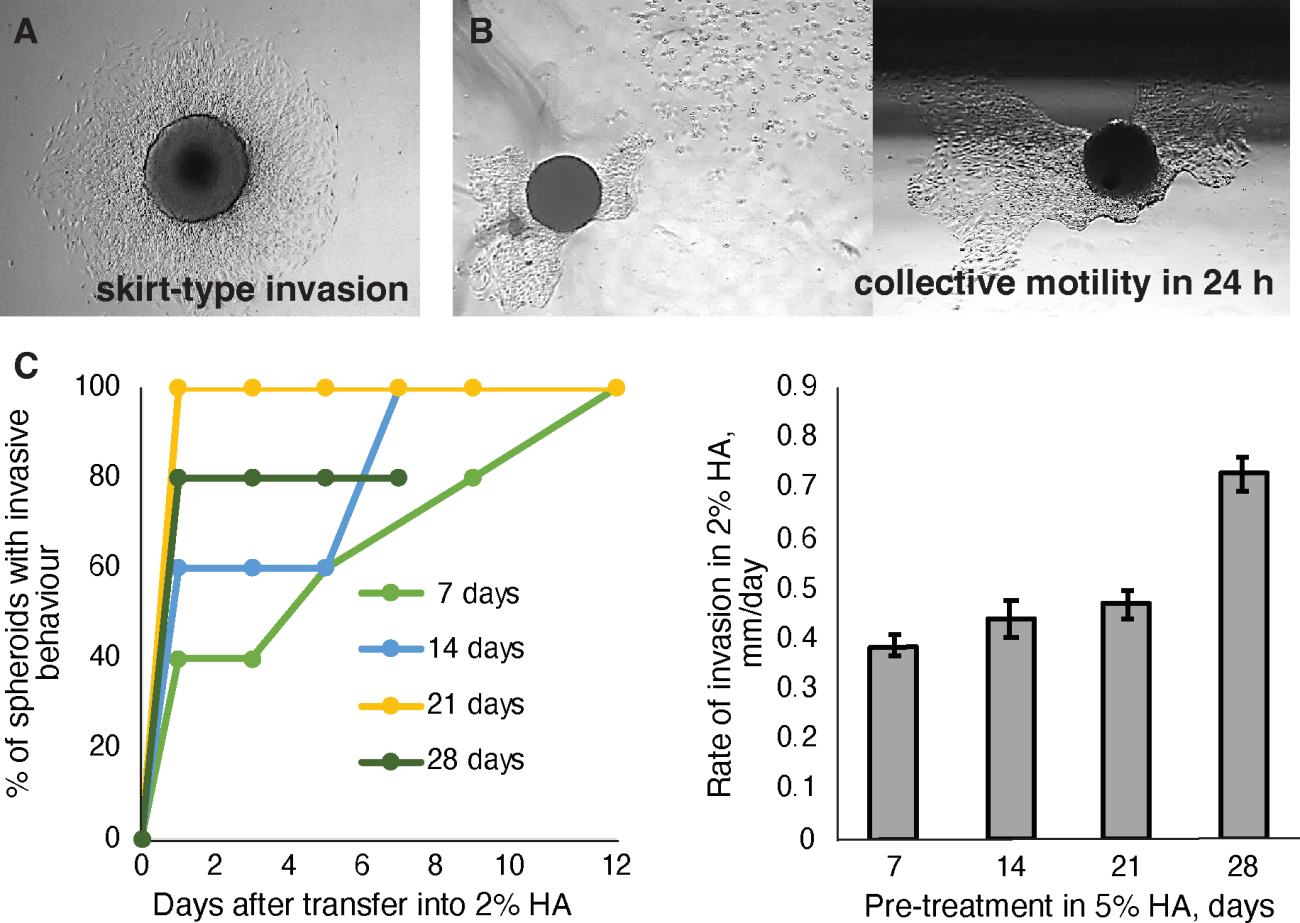
U87 spheroid dormancy and invasiveness in HMW-HA. Spheroids were cultivated in liquid medium for 7 days and placed into HMW-HA. (A) Typical skirt-type invasion inside 2 wt% HMW-HA. Invasion occurred on day 1 after spheroid placement and continued as a gradual increase in ‘skirt’ radius. By day 7−10 spherical structure became invisible, i.e. full cell dispersion was achieved. Field view 1900 µm x 2500 µm. (B) Collective cell motility inside 2−3.5 wt% HMW-HA. Sometimes a cellular sheet of an irregular form appeared around a spheroid on day 2−3 after the spheroid was placed in HMW-HA; after 24 h the spheroid and cellular sheet were found several millimetres away from their initial position, for example, next to the well wall (black shadow on the top of right picture). Dispersion of single cells was also observed, and their movement was independent from the cellular sheet and spheroid. Field view 1900 µm x 2500 µm. (C) Invasion into 2 wt% HMW-HA after a period of dormancy in 5 wt% HMW-HA. Spheroids were placed inside 5 wt% HMW-HA for 7, 14, 21 and 28 days (five spheroids per each period of dormancy). During dormancy period spheroid shape and diameter did not change, and no cell dispersion was observed. When spheroids were transferred into 2 wt% HMW-HA the percentage of spheroids able to disperse cells (‘invasive spheroids’) was calculated (left). Rate of invasion was calculated as an enlargement ‘skirt’ radius per day (right). Error bars represent SE.

Another noticeable cell morphological feature, which was present in 2–3.5 wt% HMW-HA, but absent in 5% HMW-HA, was a collectively moving ‘cellular sheet’ (figure 4B). It was previously shown that collective cell motility (i.e. mass migration of cancer cells in sheets, strands and clusters) results in a very rapid invasion in comparison with individual cell movement, but requires maintenance of cell–cell junctions [47]. For example, elevated expression of desmoplakin and plakoblobin—the two most abundant proteins in desmosomal junction—facilitate breast and lung cancer cells to form clusters and increase their survival in circulation [48]. In 5 wt% HMW-HA, no proteins from any type of cell–cell junction were detected (figure 3C), but in 2 wt% HMW-HA, all desmosomal cadherins and linker proteins were present with desmoplakin and plakoglobin being the most upregulated (figure 3C). Proteins from other junctions were also present, for example α-catenin from adherens junction and ZO-1 from tight junctions, but only the desmosomal proteome was complete (figure 3C). Hence, we can speculate that the ‘cellular sheet’ observed in 2–3.5 wt% HMW-HA (figure 4B) was held together primarily by desmosomes.

Based on the expected changes observed in invadopodium and cell–cell junction proteome at different HMW-HA concentrations, we considered these samples representative for a pilot detection of proteins which underpin physiological changes in U87 cells. The major physiological change which occurred in 5 wt% HMW-HA compared with 2 wt% HMW-HA was spheroid dormancy: cancer spheroids placed in 5 wt% HMW-HA did not grow, invade or die, but remained dormant until transferred into to 2 wt% HMW-HA (figure 4C). In 5 wt% HMW-HA, the expression of 193 proteins was increased > 4 fold and 138 proteins were synthetized *de novo* compared with 2 wt%-HMW HA (see electronic supplementary material, table S3), thus are likely to be involved in quiescence uphold in U87 cells. According to GO annotation, these proteins are involved in biological processes essential for cell survival and metabolism support, such as: chromosome packaging, ribosome hibernation and biogenesis, mitochondria maintenance, cellular response to starvation, apoptosis suppression, tumour acidosis, glucose storage, activation of hexosamine pathway, peptide metabolism, protein biosynthesis and control of folding, proteolysis regulation, and protein translocation and trafficking (see electronic supplementary material, table S3).

Substrate mechanics affects cell behaviour transition from proliferation to dormancy [49], and is coordinated by intracellular signalling with the Notch pathway being widely recognized as a major determinant of cell fate across all metazoans [50]. In our data, Notch-2 was strongly upregulated for spheroids in 5 wt% HMW-HA and below detection in 2 wt% HMW-HA after total cell dispersion from the spheroid (figure 3C). Notch-1 was not detected in either of the samples. Hence, similar to adult brain where Notch-2 was shown to control quiescence of neuronal stem cells [51,52], Notch-2 may convey quiescence of U87 cells in 5 wt% HMW-HA. Search for an HA receptor in our model yielded only one protein—CD44, which was upregulated following the increase in HMW-HA concentration as expected for a chemoreceptor (figure 3C). The CD44 and Notch signalling pathways share two key proteins: γ-secretase, which cleaves intracellular domains known as CD44-ICD and NICD respectively [53–55] and dynamin, which enables formation of endocytosis vesicles essential for nucleus translocation of both domains [56,57]. Indicative of activation of the CD44/Notch axis during dormancy in 5 wt% HMW-HA, dynamin-2 and nicastrin (part of the γ-secretase complex) were detected for spheroids in 5 wt% HMW-HA, but not in 2 wt% HMW-HA (figure 3C). Two regulators of the Notch signalling pathway—osteonectin (SPARC) and vitamin K-dependent protein S (PROS1) [58,59] were also upregulated in 5 wt% HMW-HA (figure 3C), again indicative of Notch involvement in U87 cell behaviour determination in 5 wt% HMW-HA.

Only one hyaluronidase was detected in our samples—cell migration-inducing and hyaluronan-binding protein (CEMIP). However, its concentration was extremely low, remaining below the detection limit in all samples except one, where it was detected only in trace amounts (figure 3C). Thus, given that the only difference between spheroids in 2 and 5 wt% HMW-HA initially was the HA concentration and that the only abundant cell surface receptor for HA in our proteomics data was CD44 (figure 4C), the differences in cell morphology and behaviour (figures 1,3 , 3 and 4) were likely to be the result of differences in HA-CD44-mediated signalling and correlate with the nanosecond time scale conformational flexibility of the HA polymers discovered by NMR spectroscopy (2 wt% HMW-HA) or relative lack of such molecular flexibility (5 wt% HMW-HA). This supports our hypothesis that CD44 signalling is induced by HA molecules that are flexible on the nanosecond time scale and that in turn depends on whether the HA molecules are strongly or weakly bound into the ECM.

### 2.5. Immobilization of HA inhibits cancer cell migration and alters expression of signalling proteins similarly to 5 wt% HMW-HA

We reasoned that if flexible HA molecules promote CD44 signalling, then chemically crosslinking such molecules into the ECM should substantially decrease their molecular flexibility, and result in similar inhibition of CD44 signalling to that observed in 5 wt% HMW-HA. To test this, we synthesized a crosslinkable HA polymer, oxidized hyaluronic acid (oxHA) where 35% of the glucuronic acid rings are oxidized resulting in two aldehyde groups on the oxidized ring (see electronic supplementary material, Materials and Methods and figure S1 for details of oxHA characterization). Hence, most of the oxHA molecule had the same composition and structure as native HA, but its aldehyde groups on the oxidized glucuronic acid rings could rapidly crosslink to terminal amine groups of proteins in the ECM and thus significantly decrease its flexibility.

We then repeated our cell migration study and proteomics measurements for U87 spheroids in a 50: 50 by weight mixture of 2 wt% HMW-HA and 2 wt% oxHA. The oxHA must have the same or lower molecular weight than the original HMW-HA. Thus, 2 wt% oxHA was expected to drive cancer cell migration similarly or more rapidly than 2 wt% HMW-HA, if molecular weight were the crucial parameter in HA-cell signalling. However, we found that there was no migration in the oxHA-HA mixture.

We hypothesized that the arrest of invasion caused by oxHA was the loss of molecular flexibility in the HA-chains cross-linked to ECM proteins. The reduced molecular flexibility of oxHA-chains was expected to cause changes in protein expression as in 5 wt% HMW-HA though HA concentration remained 2 wt%. Indeed, we found that cells in 2 wt% oxHA-HA gel expressed the same Notch signalling pathway proteins as in 5 wt% HMW-HA (table 1). Furthermore, other proteins important for Notch signalling, such as ADAM proteases involved in extracellular cleavage of Notch and sugar transferases required for Notch glycosylation [60] were additionally detected in oxHA-treated sample, implying that regulation of cell behaviour in 2 wt% oxHA-HA gel was depend on the Notch pathway even more than in 5 wt% HMW-HA gel.

**Table 1.** Detection of proteins involved in Notch signally pathway in 2 wt% HMW-HA, 5 wt% HMW-HA and 2 wt% oxHA/HA mixture by shotgun label-free proteomics.

protein	type of HA mixture		
	oxHA/HA	5 **wt% HA**	2 **wt% HA**
notch-2	present	present	absent
dynamin- 2	present	present	absent
nicastrin	present	present	absent
vitamin K-dependent protein S (PROS1)	present	present	present
osteonectin (SPARC)	absent	present	absent
EGF domain specific O-linked N-acetylglucosamine transferase (EOGT)	present	absent	absent
o-glucosyltransferase 1 (POGLUT1)	present	absent	absent
glucoside xylosyltransferase 1 (GXYLT1)	present	absent	absent
protease ADAM10	present	absent	absent
protease ADAM17	present	absent	absent

## 3. Discussion

HA-CD44 signalling is initiated by close intermolecular contact (binding) of an HA molecule segment approximately 6–8 saccharide rings in length within the binding pocket in the CD44 HABD [20,21,28,29]. For this binding to occur, several processes have to happen: the relevant segment of the HA molecule must detach from its ECM intermolecular contacts and the binding HA segment adapt its conformation to that required for strong binding in the CD44 HABD [20]. These two processes must occur on a time scale which is faster than or similar to the typical time taken for the CD44 HABD to change its conformation to allow strong HA binding, which from molecular dynamics calculations is a few 100 nanoseconds [28]. We have shown here through measurement of ^1^H NMR relaxation times that dilute HMW-HA molecules as well as LMW-HA molecules have the nanosecond time scale flexibility needed to achieve strong binding to the CD44 HABD. We found cancer cell migration in both dilute HMW-HA and LMW-HA, but cell migration was inhibited in higher concentration HMW-HA gels which ^1^H NMR measurements showed did not have sufficient molecular flexibility to achieve strong binding to the CD44 HABD. When we blocked CD44-HA binding with a polyclonal antibody, cancer cell migration was significantly delayed, showing that strong CD44-HA binding is important for cancer cell migration. Our results collectively suggest that the important parameter for HA in cancer progression is its molecular flexibility for binding to CD44, rather than HA molecular weight. It is clear from this study that long as well as short HA polymers can achieve the necessary flexibility in the right environment.

Interestingly, the viscosity of the dilute HMW-HA gel used here (2 wt%) is considerably higher than that of the LMW-HA solutions investigated, yet we find similar extents of cell migration with time for the LMW-HA and dilute HMW-HA cases. Moreover, for at least one cell line (U87), we found no cell migration in media alone, an even lower viscosity case. These data show that low ECM viscosity is not sufficient to initiate cancer cell migration, at least for the GBM cell lines studied here and confirm the importance of understanding the molecular properties of the ECM ligands that influence cancer cell fate.

To initiate invasion signalling, the CD44-HA complex that results from strong CD44-HABD binding must interact with the relevant members of the invadopodia development pathway. For these interactions to occur, CD44 must be released from cholesterol-rich lipid raft in the plasma membrane into phosphatidylinositol 4,5-bisphosphate (PIP2)-enriched region [61]. PIP2 activates the phosphorylation of CD44 and, in turn, that phosphorylation allows CD44 to bind to ERM proteins (moesin/ezrin/radixin) which activates the CD44-dependent Rho-signalling pathway and invadopodia extension [62]. Binding a hydrophilic HA molecule to CD44 residing in hydrophobic lipid raft can be expected to lower the energy for translocation into PIP2-enriched (hydrophilic) membrane regions, but translocation also requires either that the HA molecule is sufficiently flexible to allow the CD44 HABD–HA binding to persist as CD44 to moves between membrane domains, or the CD44-bound HA must readily dissociate from its ECM contacts to allow the translocation. Thus, HA flexibility and its strength of binding into the ECM are highly relevant both for the initial HA binding to the CD44 HABD and for the subsequent signalling pathways.

Our pilot proteomics data on the samples obtained from cells in 5 wt% and 2 wt% HMW-HA clearly indicates that U87 cell proteome undergoes considerable regulation of protein expression in response to the change of molecular flexibility of HA between these two HMW-HA concentrations: cell morphology, cytoskeleton structure, cell–cell junctions and overall cell physiology. Our most important finding is a possible involvement of Notch-2 and its signalling pathway (dynamin-2, nicastrin, SPARC and PROS1) in dormancy of cancer spheroids in a HMW-HA concentration (5 wt%) representative of normal brain ECM. Hence, similar to adult brain where Notch-2 was shown to control quiescence of neuronal stem cells [51,52], Notch-2 may convey quiescence to brain cancer cells when HA molecular flexibility is low.

Increased HA molecular flexibility can be expected wherever there is a reduction in HA concentration through increased water content of a tissue or where the HA network is disrupted by other ECM components. Oedema as a result of brain tumour or surgical resection can be expected to dilute HA in the tumour margins potentially initiating invasion signals for remaining cancer cells; significantly, it is in these same regions where tumour re-growth is commonly seen. Infiltration of the brain extracellular HA/glycosaminoglycan network by extracellular matrix proteins expressed by cancer cells or cancer-associated fibroblasts is another potential scenario that may increase the probability of HA molecules dissociating from the ECM during their thermal motions, and so the conditions under which cell signalling is initiated. Interestingly, Safarians *et al.* [17] found that the HA concentration which maximized patient-derived GBM cell migration was dependent on the cell line, whilst other work shows that HA interacts primarily with the cell glycocalyx and more rarely with CD44 [63]. Taken together, these works suggest that HA–CD44 interactions are finely tuned by the cell surface and that the cell glycocalyx is likely to be an important further moderator of HA molecular flexibility.

HA degradation into lower-molecular-weight fractions is a commonly observed scenario in tumours [5,64–67] and can be expected to result in more flexible HA molecules, as we have shown here, because of the lower degree of polymer entanglement for shorter molecules. HA degradation is frequently correlated with poor patient prognosis and disease progression [65,66], which we speculate is related fundamentally to the increased flexibility of lower-molecular-weight HA leading to a higher probability of strong HA–CD44 binding events, and concomitant CD44-mediated cell signalling.

## Data Availability

All raw data can be found at: https://doi.org/10.17863/CAM.118962. Supplementary material is available online [68].

## Footnotes

^1^
Rheology (electronic supplementary material, figure S4) shows that the HMW-HA preparations used in this work are viscoelastic liquids. However, they do not flow when inverted and so we refer to them throughout as ‘gels’.
